# Computer Vision for Autonomous Drill Jumbos: Detecting Non-Drillable Areas of a Mine Face

**DOI:** 10.3390/s26092623

**Published:** 2026-04-23

**Authors:** Moritz Rösgen, Adam Pekarski, Moritz Ziegler, Elisabeth Clausen

**Affiliations:** Institute for Advanced Mining Technologies (AMT), RWTH Aachen University, 52062 Aachen, Germany; apekarski@amt.rwth-aachen.de (A.P.); mziegler@amt.rwth-aachen.de (M.Z.); eclausen@amt.rwth-aachen.de (E.C.)

**Keywords:** stereo vision, machine learning, computer vision, automation, mining, drill jumbo, drill and blast, underground mining, blast hole, drilling

## Abstract

The mining industry is in need of automation due to increasing requirements like higher global demands for resources and deposits, which are deeper and more complex. Progressing underground mines lead to longer travel times to the mining face and thus a loss in productive working time, which has to be compensated by automation. Ultimately, stricter health and safety regulations and a decreasing number of skilled operators accelerate the need for automation further. Within the the drill-and-blast cycle in underground mining, the drilling of blast holes is a central step. While semi-automated and supporting systems exist that allow the automated execution of single process steps under supervision, to date, no system is available for the unsupervised blast hole drilling of a mine face. A precondition for unsupervised operation is a perception system, which allows independent decision-making of the machine. To address this gap, this work presents a novel vision system capable of segmenting a mine face into drillable and non-drillable areas, which can serve as the basis for the autonomous adaption of a drilling pattern. An area of the mine face is considered drillable if no leftover blast holes from the previous blast cycle are present and the surface angle is below a certain threshold. The system presented is based on a stereo camera setup mounted on a drill jumbo. The resulting 2D and 3D data are processed by software that employs AI-based computer vision techniques, as well as traditional algorithms. The system was validated, and the performance was verified in the K+S Zielitz mine. Experts assisted in the determination of operational parameters and empirically validated the system’s performance. Additionally, the blast hole detection algorithm underwent a data-based analytical verification.

## 1. Introduction

Drilling of blast holes as a prerequisite for subsequent charging and detonation is a crucial step of the drill-and-blast cycle in underground mining. The accurate execution of the blast hole drill pattern is essential for a good blasting result to ensure both a proper fragmentation of the rock and a planned and controlled advancement of the mining face. Drilling of blast holes is a time-consuming step in the mining process that has to be carried out diligently and with precision. Consequently, automation of this process step can increase efficiency. The unsupervised execution of blast hole drilling makes the operator available for other tasks or allows drilling during shift changes, which effectively increases the available net working time at the mine face. Simultaneously, an automated system ensures the quality of the blasting result.

Currently, the drilling process for blast hole drilling is often carried out in a semi-automated manner. Numerous systems offer assistance to the operator in the drilling process like parallel boom holding, internal collision avoidance, pre-setting of drilling angle and depth, or visualization of the drill pattern. Some more advanced systems are able to drill a sequence of holes according to a predetermined pattern, requiring no or minimal involvement of the operator [1,2].

However, while being a significant improvement on productivity and workplace quality, modern assistance systems still rely on operator supervision throughout the drilling process. While, in theory, an entire blast hole pattern could be drilled unsupervised by such a system, in practice, this is almost never the case. Irregularities in the mining face result in non-drillable areas (NDAs), where blast hole drilling is impractical, unsafe, or near impossible due to the geometry or other characteristics of the rock. Developing a truly autonomous drilling jumbo therefore requires a perception system that can detect NDAs at the mining face.

In this work, a novel perception system based on stereo vision for the three-dimensional detection of NDA at the mine face is presented. Utilizing the unique advantages of stereo vision, we leverage both image processing of the stereo image and geometrical analysis of the point cloud in order to develop an advanced perception system for drill jumbos. The development of the system was realized in cooperation with and financed by the german mining company K+S AG. It was developed as part of a superordinate process for the custom-tailored automation of the drill jumbo [3]. Demonstration and validation were conducted in the underground potash mine Zielitz in Saxony-Anhalt, Germany.

In Section 2, the drilling process in underground mining is described. Subsequently, the problem is laid out, and the resulting requirements for a perception system are derived. Considering the requirements, Section 3 provides a brief overview on related work in the field of autonomous drill jumbos and evaluates the suitability of existing systems for the given problem. In Section 4, the developed system is described. Section 5 presents the results which were achieved in underground testing and Section 6 concludes this work.

## 2. Operational Background and Problem Statement

With an annual production of approximately 12 million metric tons of potash, the Zielitz mine is one of the largest potash mines in the world [4]. The mine is operated in room-and-pillar mining with drill-and-blast methods in a three-shift system. As mining operations progress and the deposit is depleted, the outer production areas are increasingly moving away from the shafts as the single point of entry. This results in a decreasing net working time in the outer areas of the mine, which leads to a production deficit that has to be compensated by automation. With its first production starting in 1973 [4], the outer areas of the Zielitz mine have, over time, moved away significantly from the shaft.

**Figure 1:**
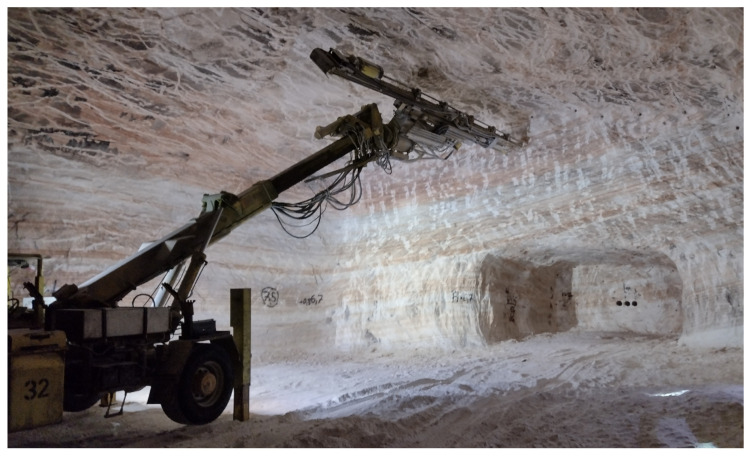
Drill jumbo set up in working position at the Zielitz mine while drilling the overhead section.

In many mining operations, including the Zielitz mine, the drilling of blast holes in the drill pattern is semi-automated. Figure 1 shows the drill jumbo during the drilling process. In this case, the jumbo is positioned approximately 9 m from the working face. To set up the drill jumbo in working position, the stakes are extended, and the boom is extended from the transport position to the face.

**Figure 2:**
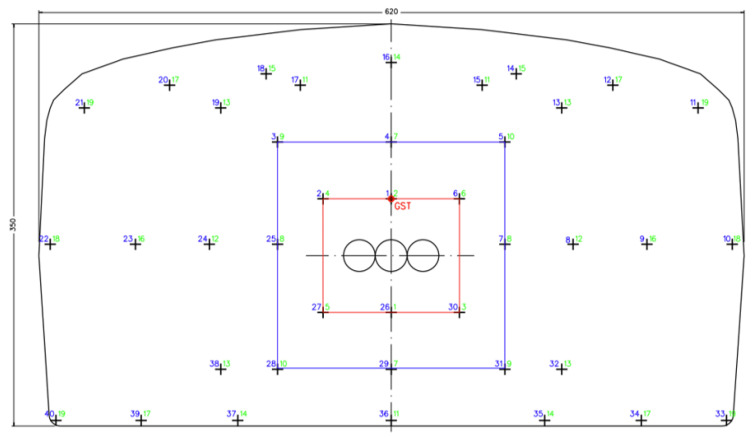
Drill pattern of the advance section at the Zielitz mine. The blast hole at the GST position (marked in red) is drilled first and determines the pose of the entire 40-hole pattern. The red and blue crates show the first- and second-shot ignition stage, respectively, with the remaining blast holes being blasted in the third stage.

**Figure 3:**
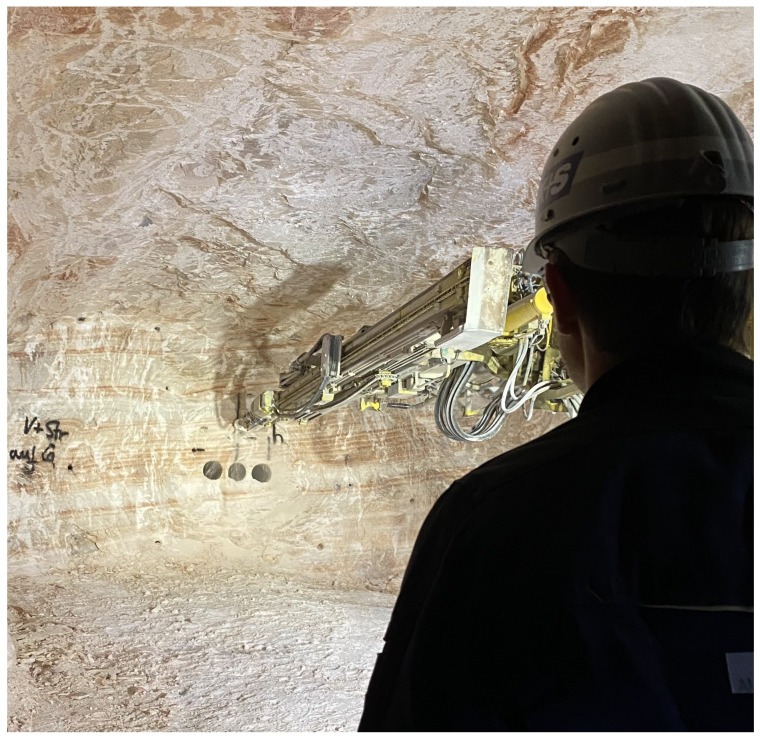
Drill jumbo from the operator’s view, immediately before drilling the first blast hole. The boom is extended to GST position above the large bore holes in the advance section. During the following drilling of the pattern, the operator is supervising the drilling process and monitoring for NDAs.

Figure 2 exemplarily shows the drill pattern for the advance section in the Zielitz mining process. In Figure 3, the boom of the jumbo is initially set to the first blast hole (marked GST, “Grundstellung” in Figure 2). The position and orientation of GST, in turn, are determined by three larger boreholes with a diameter of 280 mm, with GST located 60 cm above the center of the middle large borehole. The large boreholes are drilled in a previous step of the drill-and-blast cycle and serve as the break-in area during blasting. All blast holes in the drill pattern are known relative to the GST location. Thus, the desired three-dimensional position and orientation of each individual blast hole in the drill pattern is known relative to the jumbo after the setup procedure. The holes can then be approached automatically by the drill jumbo using the inverse kinematics of the rig. Operator intervention is required if there is a risk of the rig colliding with the surroundings or if the blast hole falls into an NDA. Collisions with the surroundings are particularly likely when drilling the ridge and base holes of the pattern, as the drill jumbo is moved closely to the roof and floor. Collisions with the mining face are less likely while drilling the inner holes of the drill pattern. In the event that the blast hole falls into an NDA, the drilling starting point is adjusted manually in a rule-based manner so that the blast hole can be drilled safely. The new starting point for the blast hole is manually set to a new location, which can deviate up to 20 cm from the originally intended starting point. In particular, when drilling the inner boreholes of the pattern, the operator’s main task is therefore to avoid the NDA and to adjust the drill pattern accordingly for guaranteeing good blasting results.

### 2.1. Non-Drillable Areas

**Figure 4:**
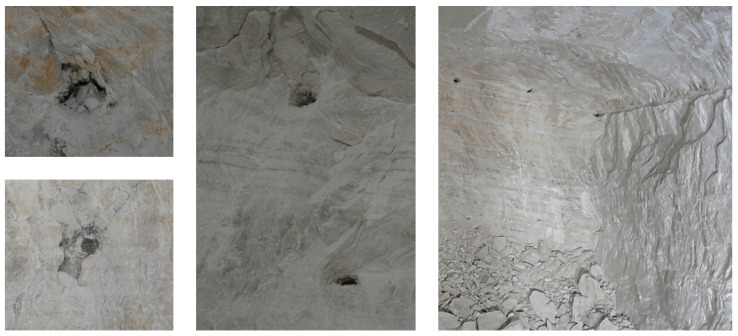
Boxes shown in close-up and at a distance on the mining face.

During potash mining at the Zielitz mine, various types of NDA occur with varying frequencies and must be avoided during the drilling process. So-called boxes are remnants of blast holes from the previous blasting cycle that were not completely blasted out and are therefore visible on the face. Figure 4 shows boxes in different variations in detail (left) and at the mine face (right). As can be seen on the right, the boxes appear as black spots from a distance and are the residuals of the upper blast holes of the pattern (see Figure 2). It is apparent that boxes can generally be detected optically.

Drilling in proximity to a box can cause the blast hole to deviate, as the drill string might follow the course of the box (meaning the previous blast hole). At the deepest point of the blast hole, the intended arrangement of the boreholes according to the drill pattern is therefore not achieved, which might impair the blasting result. In the worst case, the drill string can jam during uncontrolled passage through the box, which can lead to loss of the drill string and thus to considerable damage to the equipment and delays in operation. Boxes occur as NDAs in almost every cut. Since the drill patterns of two consecutive blasts are usually identical, boxes are more likely to occur in the area of the intended drill holes. For the automation of the drill jumbo, boxes must therefore be reliably detected while some amount of false detections with no actual underlying boxes are tolerable.

**Figure 5:**
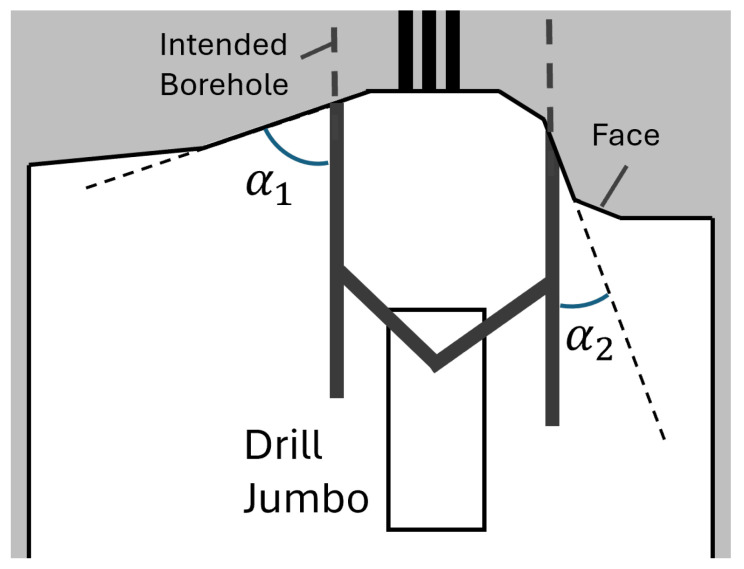
Slopes on the mining face that can cause the drill bit to slide off.

Slopes and bevelled areas in the working face can represent NDAs if their steepness exceeds the tolerable level. If there are very acute angles between the drill string and the surface of the working face, the drill bit may slip at the beginning of the drilling process, preventing controlled drilling and thus a good blasting result. Figure 5 displays the angular relations between drill and face. Within limits and based on their experience, the operator compensates for the steepness of the face by drilling slowly. If the limit angle α is steeper than a threshold angle, the surface cannot be drilled. Slopes in the working face occur frequently and must therefore be reliably detectable by automated means. In contrast to boxes, slopes cannot be easily detected by visual features in a two-dimensional image.

In addition, there are special cases that are considered NDA or prevent the drilling of the entire hole. These include, for example, exploration drill pipes that have remained in the blast hole in a compressed state and must be avoided as NDAs. Other special cases are so-called failures, i.e., unused residues of explosives in a blast hole from the previous drill hole, which completely prevent the drilling of the new drill hole. Since these are very rare and complex special cases that, as in the case of residual explosives, pose a high safety risk, manual intervention is required here. Therefore, the automated detection of these special cases is not pursued.

### 2.2. Requirements

**Figure 6:**
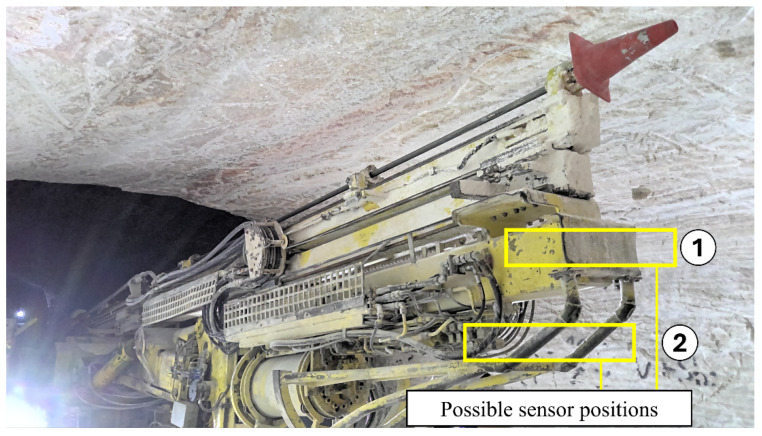
Position 1 and 2 are the possible sensor positions on the boom.

In addition to its detection capability, it needs to be possible for the sensor system to be integrated into the drill jumbo. During the drilling process, the boom is extended to the face, as can be seen in Figure 1. During this phase, no visual inspection of the face is possible, as the boom itself would block the sensor view. Consequently, the face must be recorded before the drilling operation is started while the boom is not yet extended. This is possible since the jumbo is hydraulically set up on stakes and is not moved after the initial setup. Figure 6 shows possible sensor positions, which provide equally adequate and unblocked views to the mine face while still being protected against collisions during the drilling process. In this situation, the distance of the sensor to the working face is approximately 9 m.

As mining environments often present adverse environmental conditions for optical sensors like dust and moisture, these have to be taken into consideration during the requirement definition. Due to the nature of the potash deposit, there is generally no freely available moisture in this specific operation environment. In Figure 6, however, it can be seen how potash, under the influence of drilling fluid, has stuck to the upper part of the carriage. It is therefore necessary to protect an optical sensor from similar influences. Contrary to that, airborne dust is not a critical performance limitation for optical sensors during this process step in the given operation environment at the Zielitz mine. Because only one single recording of the mine face is taken before the start of the drilling operation, there is not yet significant dust emission during the recording time. Thus, exposure of the senor to the environment could be minimized by designing additional protection equipment like closing shutters, exposing the sensor only at the moment of recording and protecting it during unused times.

Based on the above-mentioned description, the requirements for the developed system are defined as follows:

- Boxes and slopes must be recognized as NDAs with quantifiable reliability.
- Three-dimensional detection of the environment must be possible, as the detected artifacts must be spatially located. The detection of slopes also requires geometric perception.
- It must be possible to capture the working face optically or using imaging technology, as boxes are mainly detected visually.
- The system must be integrable into the drill jumbo and the process, meaning that it must be usable from its working position (9 m distance from the working face).
- The analysis of the face is not required in a live feed. Right after setup and before the drilling process, only a single recording of the face is recorded and analyzed. In comparison to the total duration of the setup process, a computation time of several seconds up to one minute is acceptable.

## 3. Related Work

The following section provides an overview of the current state of drill jumbo automation, focusing on perception systems with the purpose to assess the surface quality of the mining face.

Currently, several OEMs offer underground drill jumbos that are automated to various degrees. Equipment manufacturer Epiroc AB’s Simba Automation package [1] offers a range of assistance systems for underground drill jumbos such as automatic bit changing, detection of voids inside the drilled rock, or a measurement-while-drilling (MWD) diagnostics system. The majority of these features fall outside the scope of this work, as they aim to increase the drilling progress or reduce wear of the drill bit or machine. With respect to the scope of this work, the drill plan adaptation feature is the most relevant among the Simba features. Drill plan adaptation recalculates the positions of blast holes in the case of irregularities in the rock face. However, rock irregularities or NDAs are not assessed and marked by an automated system but by the supervising operator [1].

Similarly, Sandvik’s Drilling Automation Packages [2] include comparably advanced assistance systems. The most relevant is the automatic boom control feature, which enables the jumbo to drill a sequence of holes in predefined locations. In the event of uneven rock conditions, the drill plan can be changed. However, an operator is required to decide if holes are drillable and to intervene accordingly [2].

Both systems are designed as advanced teleremote solutions, which can execute isolated tasks in an automated manner, but they ultimately leave decision-making to the supervising operator. This means that autonomous drilling of an entire drill pattern is possible under ideal rock surface conditions, but the quality of the rock face is not evaluated automatically.

In academia, drilling automation has gained some attention. Kokkinis et al. [5] performed a review on automated drilling and mining technologies. In their work, the authors subsume the majority of attention in drilling automation under the label “drilling approaches”. Most of the works reviewed document research on automation for advancement of different drilling techniques like directional drilling or more advanced Rotary Steerable Drilling Systems (RSDSs). None of the more than 80 sources analyzed by Kokkinis et al. consider the development of machine vision for drill jumbos [5].

Another review is performed by Liu et al., indicating that the majority of research in drilling technology is focused on MWD and associated monitoring tasks like monitoring of blast hole quality or monitoring wear condition of drilling bit [6]. Very few sources are given on the automatic control of drilling equipment, none of which address machine vision for drill jumbos or the assessment of rock surface parameters with respect to NDAs.

Li et al. [7] propose a LiDAR-based system for drilling jumbos, that scans the contours of the mine drift and adaptively plans a path for the boom. The planned path avoids collisions with the uneven face while optimizing the traveled path length. The authors address the reality of uneven rock faces considering path planning of the boom and collision avoidance between the boom and the drift. However, no assessment regarding NDAs is made [7].

Nikolakopoulos et al. [8] present a concept for exploration drilling under inaccessible, confined or hazardous conditions. The high-level concept proposes novel designs for drilling robots, i.e., based on legged robots, to access inaccessible areas in the mine and the reorganization of the drilling process to include multiple robotic agents interacting with each other. The concept proposed is still in an early development stage and serves as basis for subsequent research efforts [8].

In the broader field of mine face and general mine surveying, a number of approaches have been proposed in the literature. Photogrammetry and image stitching are proven procedures for 3D reconstruction from images and have previously been used in underground mines. Rutkowski et al. present a procedure based on photogrammetry for the inspection of mine shaft convergence [9]. A photogrammetric setup is placed on a working platform to survey the shaft over several months, demonstrating successful convergence detection in the low cm range.

Nie et al. propose panoramic image stitching with an array of depth cameras for the reconstruction of a longwall face in underground coal mining [10]. This enables the real-time surveying of a longwall face with an achievable frame rate of up to 25 fps.

However, while these approaches yield promising results for their respective applications, photogrammetric approaches in general rely on the reconstruction of surfaces through a multitude of images from different viewpoints, which is not a feasible method for the given static setup.

LiDAR-based methods for exploration, navigation and surveying of underground mines have been proposed in numerous works in the literature.

Terrestrial laser scanning with respect to geotechnical monitoring of rock masses is a well-known approach. First studies within the scientific literature in this field date back to the early 2000s, as Bitelli et al. demonstrate the use of terrestrial laser scanning and photogrammetry for the monitoring of landslide bodies in northern Italy [11].

More recently, Ferrein et al. propose a mobile LiDAR-based mapping platform for underground works along with an architecture for point-cloud-based map generation [12].

Ren et al. propose a novel graph-based simultaneous localization and mapping (SLAM) algorithm that was developed specifically for underground mines [13]. Both works showcase the feasibility of LiDAR-based SLAM approaches for underground mapping with respect to navigation.

Fahle et al. investigate the use of SLAM-based systems specifically for geotechnical monitoring in underground mining [14]. To this end, a suite of quality metrics for SLAM in underground monitoring is developed.

While these works represent the continuous research effort in the field of underground mine surveying and mine face detection, none of these works specifically address the problem of machine vision for a drill jumbo or the analysis of mine faces with respect to blast hole drilling. As such, the issue of detecting and evaluating NDAs with regard to autonomous drill control remains unsolved.

## 4. System Design

In this section, a novel system to segment the rock face in an underground mine into drillable and non-drillable areas is presented. The proposed system consists of a stereo camera with a dedicated processing unit as the sensor’s setup. The stereo camera records time-synchronized images, which the processing unit then uses to compute a disparity map and subsequently a 3D point cloud of the photographed scene. The 2D images and the computed 3D information are then used by several algorithms to detect NDAs. The mentioned sensor setup and the algorithms will be presented in the following subsections:

- Section 4.1 Sensor: Description of the technical background of the selected sensor technology, as well as chosen technical specifications.
- Section 4.2 Algorithms: Description of employed algorithms and hyperparameters.
  – Section 4.2.1 ROI: Calculation of the regions around the future drill holes by detecting the blast holes in the left camera image and placing the drill pattern into the scene accordingly.
  – Section 4.2.2 Box Detection: Detection of the leftover boxes with a deep learning-based approach that operates on the left camera image only.
  – Section 4.2.3 Slope Detection: Detection of slopes on the mine face that exceed a certain threshold on the 3D point cloud by a geometrical approach

**Figure 7:**
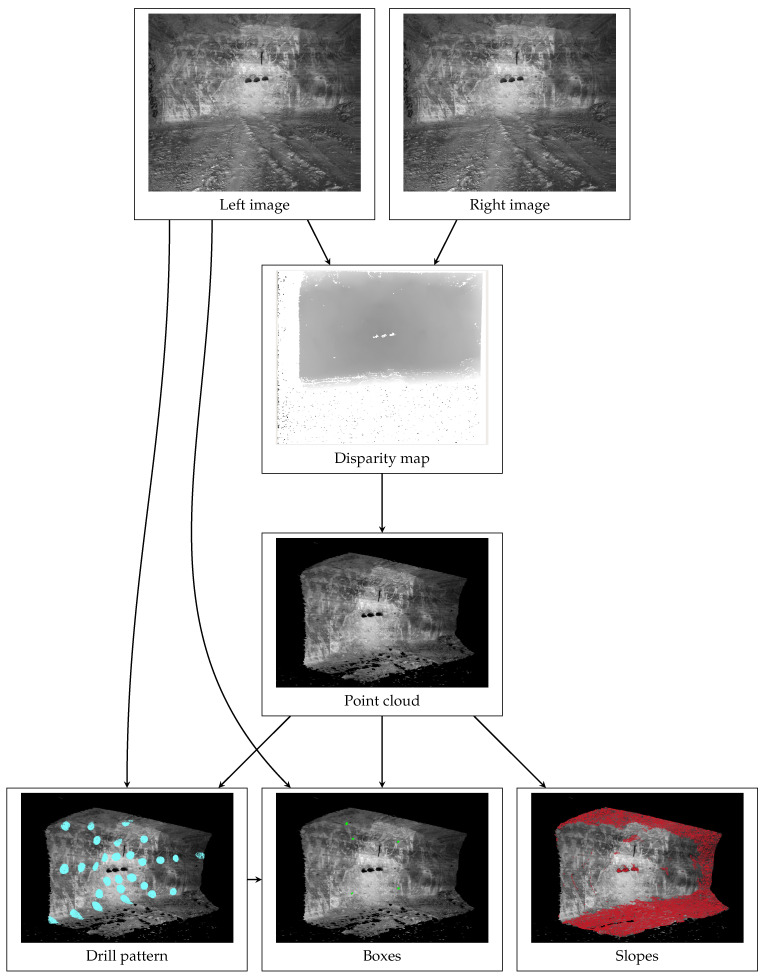
Conceptual overview of the systems’ information flow. The images from the left and right lens of the stereo camera are used to compute the disparity map. From the disparity map, the depth values for all pixels can be computed and displayed as a point cloud. The drill pattern determines the turquoise ROI for the box detection and is calculated based on the large bore holes in the left image. The box detection algorithm exclusively utilizes the left image of the stereo system for box detection. Due to the stereo relation, the three-dimensional location of the boxes can be determined in the point cloud. Slope detection is computed exclusively on the point cloud.

Figure 7 provides an overview of the computation steps performed and how they are interconnected on the basis of a representative example.

### 4.1. Sensor

Based on the requirements determined in Section 2.2, the chosen sensor system must be able to generate image and spatial information at a distance of 9 m in sufficiently high resolution to detect NDAs and spatially locate them. With these restrictions in mind, suitable sensor options are stereo vision or LiDAR–camera calibration. In this case, stereo vision was chosen because of the wide range of commercially available systems, as well as its lower complexity compared to a multi-sensor setup like LiDAR–camera calibration. Similarly to the human eyes, stereo vision uses a pair of cameras to create a pair of images, from which depth information can be derived. This makes it possible to generate a point cloud of the environment, which combines the image information of the cameras with the geometric properties of the scene. Compared to other depth camera technologies (e.g., time of flight or structured light cameras), this is a purely passive technology that does not depend on the recognition of additional light pulses.

#### 4.1.1. Technical Background of Stereo Vision

**Figure 8:**
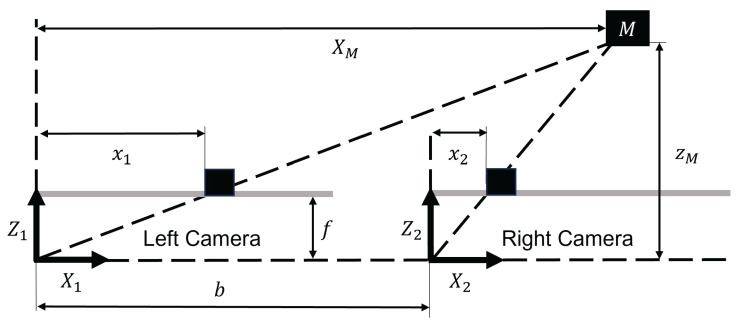
Setup of the stereo camera system.

For the reconstruction of positional information using stereo vision, a scene must be captured simultaneously by two parallel cameras. If the geometric properties of the cameras are known, the depth of a feature can be determined from its displacement (disparity) between the two images. Figure 8 visualizes a generic stereo camera setup. The baseline *b* of the system is the distance of the principal points $c_{x}$ and $c_{y}$ of the two camera sensors used. The distance between the camera lenses’ optical center and the image sensor is defined as the focal length *f*.

The difference in the feature coordinates $X_{1}$ and $X_{2}$ is called disparity:

(1) $$d=X_{1}-X_{2}$$

With the disparity *d*, the baseline *b* and the focal length *f*, the depth $z_{m}$ of the feature can be calculated using simple geometric considerations.

(2) $$z_{m}=\frac{f\;\cdot \;b}{d}$$

For the calculation of the horizontal and vertical position, the coordinates of the principal point coordinates ($c_{x}$ and $c_{y}$) are needed.

(3) $$x_{m}=\frac{(u-c_{x})\;\cdot \;b}{d}$$

(4) $$y_{m}=\frac{(v-c_{y})\;\cdot \;b}{d}$$

u and v are the image-coordinates of the corresponding pixel in the left image. Using these relations, positional information can be calculated for the majority of pixels in a given scene. The stereo-camera parameters—focal length *f*, pixel coordinates of the principal point $c_{x}$ and $c_{y}$, and baseline *b*—are inherent parameters that result from the physical design of the stereo system. However, small tolerances in the manufacturing process of the stereo system can easily result in significant deviations from the inherent parameters and thus result in a major loss of accuracy. In practice, these parameters are therefore determined in an automated fashion during the calibration procedure. This is usually done using a checkerboard pattern, as can be seen in [15].

#### 4.1.2. Parameters of the Chosen Stereo Vision System

As defined in Section 2.2, the camera system is required to have a working distance of 9 m. From this, the following requirements on the cameras themselves can be derived:

- At a distance of 9 m, the complete working face must be visible in both images.
- The camera should be focused on the working distance of 9 m.

Table 1 provides an overview of the chosen parameters of the stereo camera and stereo matching unit.

The working face has a size of approximately 6.2 m horizontally and 3.5 m vertically (Figure 2). The chosen cameras allow for the detection of a plane with the size of 8.83 m horizontally and 7.68 m vertically. This allows for the full working face being in the field of view of the camera while not recording an excess of unnecessary data. The focus of the cameras were set for a distance of 9 m using a Siemens star. The distance error of the system can be calculated using the following formula [16]:

(5) $$\begin{array}{c} \Delta z=\frac{z^{2}}{b\;\cdot \;f}\;\cdot \;\Delta d \end{array}$$

Applying Equation (5), the theoretical distance error at the working face can be approximated as

$$\Delta z\left(z=9\;m,\;b=0.54\;m,\;f=2180.08\;\mathrm{pixels},\;\Delta d=\frac{1}{16}\;\mathrm{pixel}\right)\approx 4.3\;mm.$$

According to the manufacturer’s specifications, the disparity resolution should be doubled for a more realistic distance error. Based on the information provided by the manufacturer, a disparity resolution Δd of at least $\frac{1}{8}$ is more realistic, which would yield a maximum achievable accuracy of 8.6 mm distance error. There is a trade-off between high resolution/high precision and computation time. Because the given task does not require real-time processing, a stereo system with high resolution and precision was chosen.

### 4.2. Algorithms

This subsection describes the algorithms used for detecting the leftover boxes and slopes on the mine face. An important intermediate preprocessing step is the cleanup and interpolation of the disparity map. As can be seen in Figure 7, the disparity map has holes with no values, as well as a lot of noise manifesting in visual “islands” around the main part. These islands are removed by deleting all values in an unconnected component below a certain size. Subsequently, all pixels with no values that horizontally or vertically lie between pixels with values are assigned values that are linearly interpolated. This is important since the disparity map is used to map 2D coordinates from the left camera image to 3D coordinates in the point cloud and vice versa.

#### 4.2.1. ROI According to the Drill Pattern

**Figure 9:**
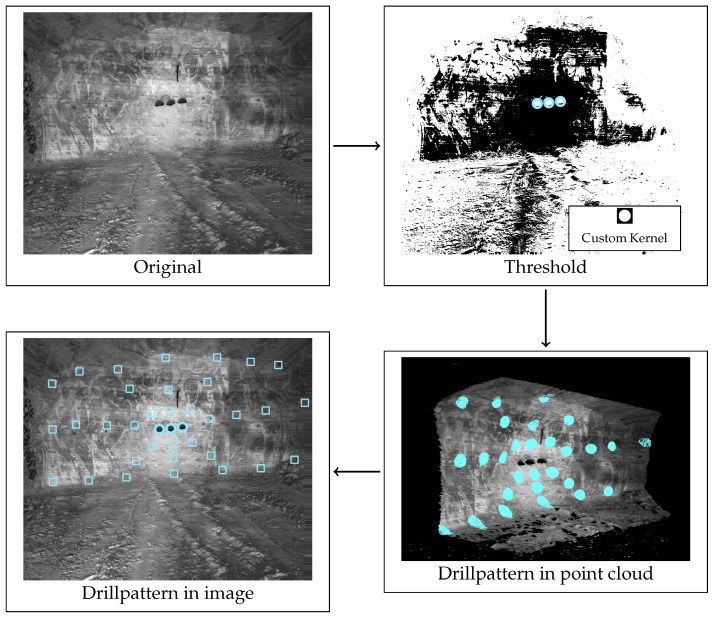
Algorithmic steps to detect large boreholes and to project the drill pattern into a scene. The blue circles in the camera images mark the detected large boreholes. The projected drill pattern is marked as blue squares in the camera image and points of the pointcloud that correspond to the drill pattern are also colored blue.

To determine drilling positions, the drill pattern is projected into the point cloud obtained by the stereo camera system. To reduce the computation time of the box detection significantly, this step is performed first, since boxes only need to be searched in the areas of intended new drill holes. Figure 9 illustrates the algorithmic steps for determining the drill pattern. The drill pattern (Figure 2) is centered around the large-diameter blast holes. In the specified drill pattern, there are three large diameter blast holes with a diameter of 28 cm. Thus, to correctly project the drill pattern onto a scene, the large-diameter blast holes must be detected first.

Because they are consistent in size and form, classical computer vision algorithms, rather than machine learning, can be used for this task. First, a binary threshold filter is applied to the image. Afterwards, a custom kernel (Figure 9) is moved over the filtered image. The custom kernel resembles the large-diameter blast holes in size and form. At each step, the average difference between the kernel and the binary image is calculated, and if it falls under a threshold, it is considered a potential large-diameter blast hole. The list of potential large-diameter blast holes is then filtered by several conditions, such as a minimum distance constraint between large-diameter blast holes.

The center of the middle large borehole has to be projected into the point cloud, because the drill pattern is spatially defined around it, and the image contains no such information. The centers of the large boreholes are then projected into the point cloud. Afterwards, the drill pattern is projected into the point cloud, based on its spatial position. Because the camera position may be tilted compared to the working face, the drill pattern needs to be rotated accordingly. A straight line is fitted through the centers of the first and third large borehole, and its angle θ is calculated in comparison to a horizontal line. Afterwards, the center of each blast hole in the drill pattern is rotated around the central large borehole using Equation (6). The rotation is only applied in a two dimensional plane, because the drill pattern is only defined in two dimensions.

(6) $$\begin{array}{cc} x^{\prime } & =x\;\cdot \;\cos(\theta )-y\;\cdot \;\sin(\theta )\\ y^{\prime } & =x\;\cdot \;\sin(\theta )-y\;\cdot \;\cos(\theta ) \end{array}$$

$$\mathrm{where}\;\;\;\begin{array}{ll} x^{\prime },y^{\prime } & \mathrm{morphed}\;\mathrm{coordinates}\\ x,y & \mathrm{original}\;\mathrm{coordinates}\\ \theta & \mathrm{rotation}\;\mathrm{angle} \end{array}$$

A blast hole may deviate a maximum of r=20 cm from its planned position. For this reason, the potential blast hole positions are projected as circles with a radius *r* around their determined centers. The positions of the drill pattern are then projected back into the image plane by rearranging Equations (3) and (4):

(7) $$u=\frac{x_{m}\;\cdot \;d}{b}+c_{x}$$

(8) $$v=\frac{y_{m}\;\cdot \;d}{b}+c_{y}$$

#### 4.2.2. Box Detection

**Figure 10:**
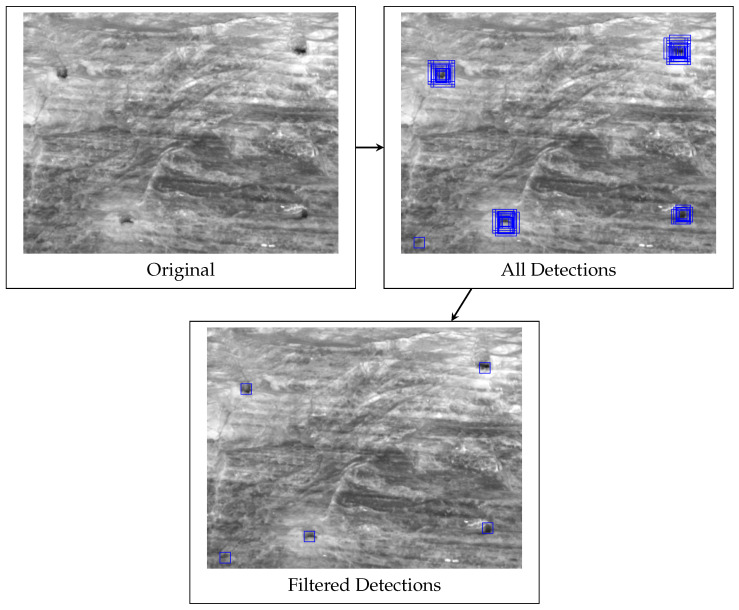
The algorithmic steps of the box detection. Four boxes are correctly identified by multiple sliding windows. One additional false positive is detected in the lower left corner. In the filtering step, the repeated detections are removed by non-maximum suppression, leaving only one marker per box in the final image. The false positive is not removed, since false positives are generally more acceptable than false negatives.

Since boxes are mainly detected visually (see Section 2.1), box detection is performed on the 2D grayscale image of the face. Because the left lens represents the origin of the stereo sensor, box detection is performed in the left camera image. This allows for easy projection of the detected boxes into three-dimensional space. To this end, a sliding window approach is paired with a deep-learning classifier followed by a non-maximum suppression filter. Figure 10 visualizes this process. The final detected boxes can be mapped to 3D positions by using the parameters of the stereo setup (see Equations (2)–(4)).

##### Sliding Window

The sliding window approach is a simple and straightforward method for object detection in images. A rectangular window of fixed size is iterated regularly across the input image. At every position, the content of the window is passed to a binary object classifier that decides if the object of interest is present in the subimage defined by the window. This process can be repeated for multiple window sizes. As seen in Figure 10, this results in multiple stacked detections for a single box. Thus, a non-maximum suppression (NMS) on the detections is performed: For every pair of detections that have an intersection over union (IoU) of at least a defined value, only the detection with a higher classifier score is kept. This results in exactly one precise detection for a box.

The sliding window approach was implemented by utilizing an image pyramid. This technique simulates increasing window sizes by stepwise decreasing the size of the whole image itself while keeping the actual window size constant. This reduces the computational cost with no disadvantages. The chosen size of iterations is 3, while the window-size is 15×15 with a stride of 2 and a factor of $\sqrt{2}$ for decreasing the size of the left image between iterations. The corresponding values for a straightforward implementation without an image pyramid and increasing window sizes would be window sizes of 15×15, 21, 21×21, 21, and 30×30 with a stride of 2, 2.828, and 4, respectively. These values were chosen based on the box-size distribution in the training data.

Using a sliding window is slow and computationally expensive, but this is not a problem since the requirements (Section 2.2) do not include any constraints for the computation time of the system. On the other hand, it allows a very fine resolution when searching for boxes inside the image and thus reduces the amount of boxes the system will not detect. Other architectures like YoLo [17] and transformer-based object detectors [18] or even non-deep-learning models like an SVM combined with a HOG [19] are viable alternatives. But evaluating these alternatives remains outside of the scope of this study, which instead focuses on establishing a baseline with the proposed binary classifier and sliding window approach.

##### DNN Object Classifier

**Figure 11:**
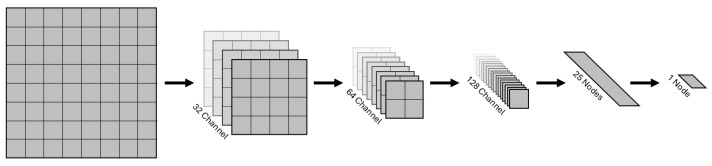
The architecture of the neural network. The first 3 arrows correspond to the convolution and pooling layers. The last 2 arrows correspond to the dense layers.

The box detection is realized using a binary object classifier that decides if a subimage contains a box or not. To this end, every subimage is transformed into an 8×8 image, which is then fed into a neural network that outputs a decimal number between 0 (no box) and 1 (box). The network architecture consists of three convolutional and max-pooling layers followed by two fully connected layers. While the input image is grayscaled and has only one channel, the output of the convolutions have 32, 64, 128 channels respectively. The kernel size is 3×3, the stride is 1, and “same” padding is used for all convolutions. The max-pool layers have a kernel of 2×2 and stride of 2. The output size of the first fully connected layer is 25. All layers have an ReLu activation function, except for the last layer, which has a sigmoid activation function. This ensures that the output lies between 0 and 1. The total amount of trainable parameters is 96,371. This architecture is a variation of LeNet [20] and can be seen in Figure 11.

To train this deep neural network, training data was collected from within the mine in Zielitz. Photos were taken from 31 different mining faces with an average of 27.71 boxes per face. The images were taken from various distances between 9 m, 11 m, and 14 m. Pictures were taken with both cameras in the stereo setup. Additionally, photos of all faces could be taken under multiple lighting conditions, of which one was the actual lights of the drill jumbo. This resulted in 698 individual pictures of mining faces.

**Figure 12:**
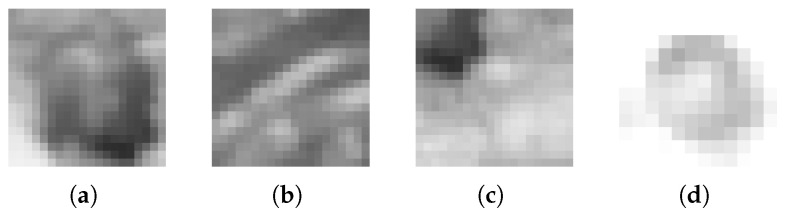
A selection of different boxes present in the training data: (**a**) a positive example: A box inside the subimage; (**b**) a negative example: no box inside the subimage; (**c**) a negative example: a small part of a box is inside the subimage; (**d**) a subimage of a box that would normally be a positive example but is overexposed and thus excluded from the dataset.

Subsequently, the boxes in all images were manually marked, resulting in 18,000 individual boxes labeled in the images used for training. Approximately 1% of these marked boxes were overexposed, which were filtered out by only allowing images with a pixel value standard deviation of at least 6. Positive examples were square images with an IoU of at least 0.6 with a box. Half of the negative examples were square subimages with an IoU of up to 0.6, while the other half were subimages from a random position on a face with no overlap of any box. Samples from the training dataset are visualized in Figure 12. The training dataset included every geometrically possible positive example (up to 30 per labeled box) and an equal number of negative examples. In total, this resulted in a dataset of approximately 420,000 positive and negative examples each, i.e., subimages containing boxes or containing no boxes.

The model was trained in PyTorch (2.5.1+cu124) [21], with the Adam optimizer [22] (*β*_1_ = 0.9; *β*_2_ = 0.999) with the binary cross entropy as the loss function and a learning rate of 0.001 Batch normalization was performed after every convolution, and dropout layers were inserted before the dense layers with a dropout probability of 0.5 After five epochs of stagnating loss, training was stopped early with no general limit to the amount of epochs. Training data was split into a standard 0.7, 0.2 and 0.1 split for training, evaluation and validation datasets, respectively. In addition to the aforementioned augmentation via translations based on the IoU, multiple forms of data augmentation, like flipping, color variations, and rotations, were used. The model’s performance on the evaluation dataset is reported in Table 2.

#### 4.2.3. Slope Detection

The slopes are detected by applying a deterministic algorithm on the point cloud provided by the stereo camera system. To reduce the computational cost, the dense point cloud is downsampled using voxelization. All points of the original point cloud in one voxel cell are combined into one voxel. This voxelization is subject to a trade-off between accuracy and computational time. The bigger the voxel cells, the less computational time is needed for the following steps. Concurrently, if the size of the voxel cells is too big, local irregularities in the mining face cannot be represented accurately enough. In this case, different voxel sizes were tested in a trial-an-error procedure to determine the largest possible voxel size that would still accurately represent the relevant slopes according to the developers domain knowledge. This decision was later confirmed by an experienced operator during the evaluation phase. As a result, the resulting voxel size is set to a cube with an edge length of 2.5 cm. Afterwards, outliers are removed by only keeping voxels that have at least n=100 points in a radius of s=50 cm around them.

**Figure 13:**
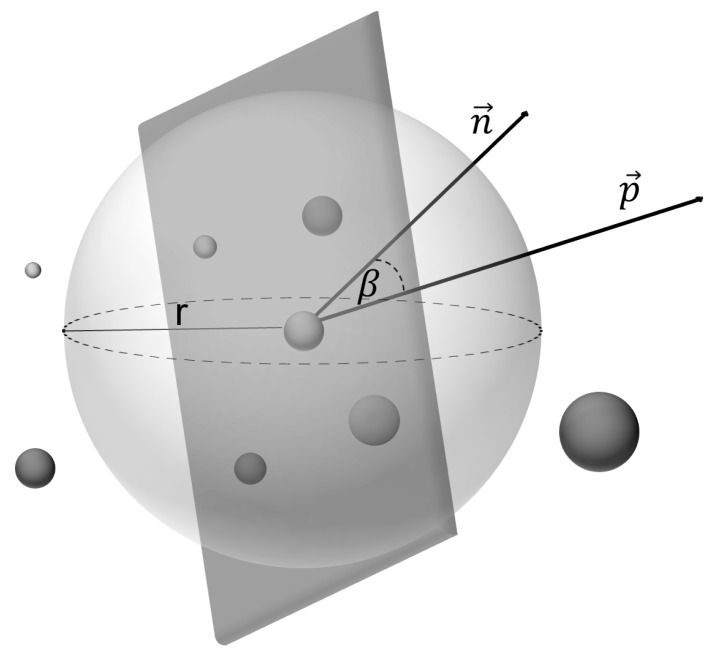
A visualization of the slope detection process. The dark gray plane represents the surface fitted through the voxels within the radius *r* (light gray sphere). The angle β is calculated between the surface normal $\vec{p}$ and the normal of the image plane $\vec{n}$.

Figure 13 visualizes the process of slope detection. Surface normals $\vec{p}$ are estimated for each voxel by fitting a plane through all voxels in a radius *r* around that voxel. The radius *r* has to be small enough to be able to map local irregularities. This value once again was determined by the described trial-and-error procedure. The resulting radius was 10 cm. This process step has the additional effect of compensating possible stereo vision reconstruction errors by considering a collection of points for the surface normal calculation. Because the stereo camera is aligned with the drilling rod, the angle between the surface normal $\vec{n}$ of the image plane and the surface normals $\vec{p}$ is determining the NDA.

To calculate the angle between the image plane and the voxel normal, the following formula is used:

(9) $$\cos\left(\beta \right)=\frac{\vec{p}\;\cdot \;\vec{n}}{∥\vec{p}∥\;\cdot \;∥\vec{n}∥}$$

The angle β is the angle between a given voxel normal $\vec{p}$ and the normal of the image plane $\vec{n}$. ∥…∥ denotes the euclidean norm, and · is the dot product. Because the surface normal of the image plane is $\vec{n}=\left(\begin{array}{c} 0\\ 0\\ 1 \end{array}\right)$ and the vector $\vec{p}$ is normalized, Equation (9) can be simplified to

(10) $$\cos\left(\beta \right)=p_{3}$$

**Figure 14:**
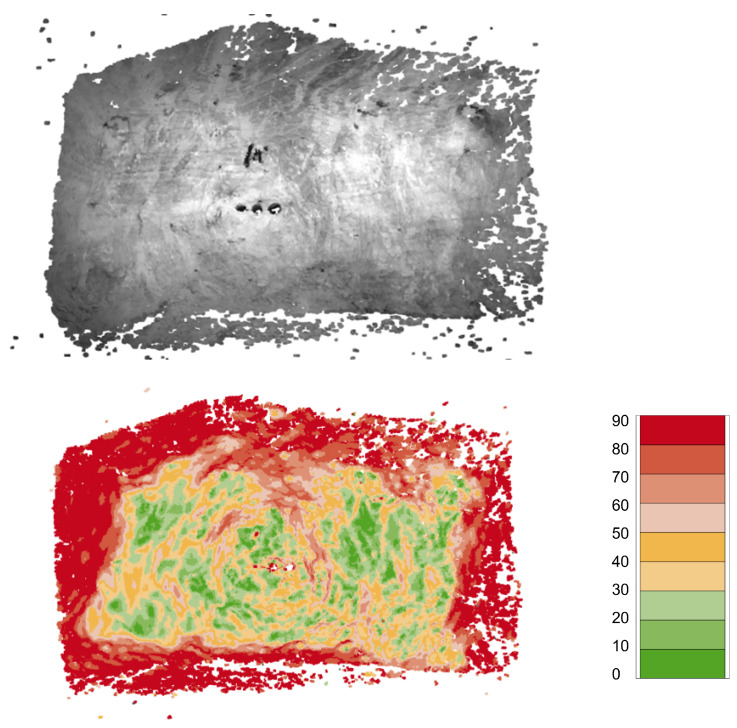
Illustration of the calculated angles for each voxel in a given frame. The color bar on the right side of the figure assigns different colors to the angle between the drill bit and the voxel normal.

Figure 14 illustrates the distribution of calculated angles for each voxel in a given frame. Note that the angle α depicted in Figure 5 is the angle between $\vec{n}$ and the fitted (dark gray) surface in Figure 13 and can be computed by

(11) α=90° − β

The cutoff angle $\beta_{\max}$ is a parameter that determines what surface angles of the rock face are considered too steep and thus are a non-drillable areas. It needs to be adjusted based on different characteristics like rock hardness, rotational speed of the drill bit, etc.

## 5. Results

Ultimately, the suitability of the system was evaluated by experienced operators during a live demonstration in the mine, while a quantitative evaluation on the recorded dataset is given later in this section. The objective of the tests was to prove the aptitude of the system, as well as parameterization of tuning parameters in cooperation with the experts. Thus, the developed system was tested rigorously in the K+S Zielitz mine (Zielitz, Germany) during development and upon completion. The performance of the stereo camera itself was examined by determining the diameter of the large boreholes, the only known geometry, in several mine faces on a random basis. The spatial error was determined to be less than 1 cm, which is sufficiently precise for the given application. The tests yielded very good results for the slope detection and box detection tasks, as operators and experts evaluated the system qualitatively based on their experience. Since these tests were not conducted using a prerecorded dataset but within a live demonstration within the mine, no quantitative analysis can be given for these demonstration cases. However, it was determined by the experienced operators that virtually all boxes were detected, with only a very small amount of undetected boxes. Visibly, these false negatives could be classified as edge cases with a very unusual shape or color. The general performance of the system was determined to be adequate for the development of an autonomous drill jumbo.

During evaluation, the crucial parameters of the algorithms were determined in cooperation with the experts. For slope detection, a cutoff angle of $\beta_{\max}=60{}^{\circ}$ was deemed optimal, as the resulting separation of the mine face into drillable and non-drillable slopes was congruent to the result based on the expertise of the operators. Additionally, for box detection, the minimum output value of the model for a window to be classified as a detected box was determined. This value was chosen to be *θ* = 0.99999, as this value resulted in nearly all boxes being detected and a tolerable amount of detections with no underlying box (false positive).

The runtime of the whole system for a given mine face is slightly less than 1 min in the mentioned tests, which were performed on a rugged laptop with an Intel Core i5-1145G7 CPU (Intel Corporation, Santa Clara, CA, USA) and no dedicated GPU. However, the majority of the computation time is attributed to the box detection process and its use of DNNs. This suggests that the runtime of the whole system could be decreased significantly using specialized hardware like GPUs to accelerate the computation of the DNNs. Note that due to the determination of the ROI based on the drill pattern, the number of windows in the sliding window approach is reduced from approximately 650,000 (for searching the entire image) to 55,000 (for searching only in the ROI). Thus, this step alone leads to a reduction in runtime of more than 90%.

**Figure 15:**
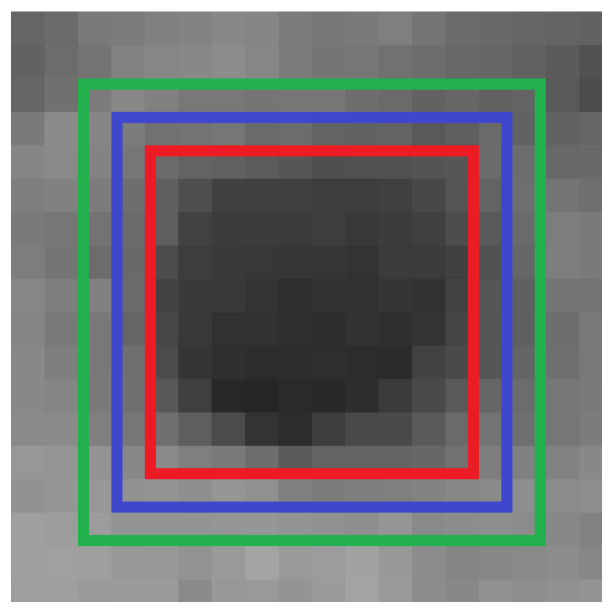
A box in the dataset with window sizes used for the validation. The blue rectangle represents the labeled ground truth. The green box is the maximal window size, and the red box is the minimal window size such that the IoU is at least 0.75.

Computing the angles of the mine face is, as discussed, a deterministic and purely geometrical approach. The quality and accuracy are exclusively dependent on the quality of the sensor outputs, the resolution of the voxelization, and the radius *r* for creating the surface normals (see Section 4.2.3). Because of that, the qualitative evaluation of the system by operators and experts is decisive for the slope detection. Contrary to that, box detection is based on machine learning, which is less deterministic and could potentially react very differently for similar inputs. Thus, the box detection was tested with a more systematic and quantitative analysis:

Because false positives are tolerable, the analysis only focuses on the amount of present boxes that are detected successfully. To this end, subimages around every box in the training dataset are taken in a sliding window approach that have an IoU with the labeling of that box of 0.75 or more with stride of 1. If at least one of these subimages gets a model score of at least θ, the corresponding box is counted as detectable by the system. Figure 15 visualizes this.

The result of this evaluation is that 97.14% of the boxes in the evaluation dataset count as detectable by the system. An underlying condition for this evaluation is that the sliding window approach is granular enough. Since real-time capability or a fast runtime are not a requirement of the system, this condition is not an issue.

## 6. Conclusions

Automation of underground drill-and-blast operations is increasingly important as mines face a shortage of skilled operators, stricter safety and health regulations, and rising productivity demands. Blast hole drilling is a particularly critical and time-consuming step that has to be carried out diligently and with precision. Consequently, automation of this process step can increase efficiency, as unsupervised execution of blast hole drilling makes the operator available for other tasks or allows drilling during shift changes. Developing such a truly autonomous drilling jumbo therefore requires a perception system that can autonomously assess the drillability of the rock face and adapt the drill pattern accordingly. In particular, there is a lack of perception systems that can reliably detect non-drillable areas (NDAs) such as remnants of previous blast holes and unfavourable surface geometries.

To close this gap, this work presents a stereo vision-based perception system that segments the mine face into drillable and non-drillable regions. An area is considered non-drillable if either leftover blast holes from the previous blast cycle (“boxes”) are present or the local surface inclination exceeds a configurable threshold angle. A stereo camera mounted on the drill jumbo captures the face from the working position, and the resulting 2D images and 3D point cloud are processed by a combination of deep learning and geometric algorithms. First, the future drill pattern is projected into the scene by detecting large-diameter reference boreholes and defining regions of interest around all planned blast holes. Within these regions, boxes are detected in the left camera image using a convolutional neural network and a sliding-window scheme, while slopes are identified directly in the voxelized point cloud by estimating local surface normals and applying an angular cutoff criterion.

The system was implemented and evaluated in underground trials at the K+S Zielitz potash mine. Experienced operators assessed the resulting segmentation as suitable for practical use and confirmed that almost all relevant NDAs were correctly identified while the system fulfilled all requirements. A quantitative analysis on a labelled dataset from 31 mine faces, resulting in more than 18,000 individual boxes, showed that 97.14% of annotated boxes were detectable with the trained model. These are significant results indicating that the proposed perception system is technically feasible for integration into autonomous drill jumbos and constitutes an essential building block towards unsupervised blast hole drilling. Next steps include extending the system to find and optimize the actual positions of the blast holes inside the drillable areas and integrating the system into the control of the drill jumbo.

## Figures and Tables

**Table 1:** Relevant parameters of the stereo system.

Parameter	Value
Focal length (physical)	6 mm
Opening angle (horizontal)	58.72°
Opening angle (vertical)	50.50°
Resolution	2464×2064
Baseline	0.54 m
Disparity resolution	$\frac{1}{16}$ pixel

**Table 2:** Evaluation metrics for the model. A threshold of 0.5 is used to classify the model’s outputs into detections and non-detections. All values are rounded to 4 decimal places.

Metric	Score
Accuracy	0.9897
Precision	0.9863
Recall	0.9928
Specificity	0.9866
F1-Score	0.9896
MCC ^1^	1.0000

^1^ Matthews correlation coefficient.

## Data Availability

The datasets presented in this article are not readily available because this study was privately funded. Requests to access the datasets should be directed to K+S AG.

## Abbreviations

The following abbreviations are used in this manuscript:

NDA	Non-Drillable Area
GST	Grundstellung
OEM	Original Equipment Manufacturer
MWD	Measurement While Drilling
ROI	Region of Interest
DNN	Deep Neural Network
NMS	Non-Maximum Suppression
IoU	Intersection over Union
